# Diversity of Mycotoxins Produced by *Fusarium* Strains Infecting Weeds

**DOI:** 10.3390/toxins15070420

**Published:** 2023-06-28

**Authors:** Sigita Janaviciene, Eimantas Venslovas, Grazina Kadziene, Neringa Matelioniene, Zane Berzina, Vadims Bartkevics, Skaidre Suproniene

**Affiliations:** 1 Department of Plant Pathology and Protection, Institute of Agriculture, Lithuanian Research Centre for Agriculture and Forestry, Instituto al. 1, Akademija, LT-58344 Kedainiai, Lithuania; eimantas.venslovas@lammc.lt; 2 Department of Soil and Crop Management, Institute of Agriculture, Lithuanian Research Centre for Agriculture and Forestry, Instituto al. 1, Akademija, LT-58344 Kedainiai, Lithuania; grazina.kadziene@lammc.lt; 3 Microbiology Laboratory, Institute of Agriculture, Lithuanian Research Centre for Agriculture and Forestry, Instituto al. 1, Akademija, LT-58344 Kedainiai, Lithuania; neringa.matelioniene@lammc.lt; 4 Institute of Food Safety, Animal Health and Environment “BIOR”, Lejupes Iela 3, LV-1076 Riga, Latvia; zaane.berzina@gmail.com (Z.B.); vadims.bartkevics@bior.lv (V.B.)

**Keywords:** *F. graminearum*, *F. avenaceum*, mycotoxins, weeds, HPLC-MS/MS

## Abstract

Although *Fusarium* is mainly known as an agricultural pathogen that affects monocotyledonous plants, it can also infect different species of weeds in the agricultural environment, thereby contributing to the production of mycotoxins in cereals. In this study, we present new developmental data on the diversity of mycotoxins produced by *Fusarium graminearum* and *Fusarium avenaceum* strains from weeds under field conditions. Regarding the potential for the strain dependence of mycotoxin production, this study demonstrated that all *F. graminearum* strains isolated from weeds and spring wheat showed high potential for deoxynivalenol (DON), 3-acetyl-deoxynivalenol (3-ADON), 15-acetyl-deoxynivalenol (15-ADON), and nivalenol (NIV) production in spring wheat under field conditions. It was determined that *F. graminearum* is a typical producer of B-type trichothecenes. All strains of *F. avenaceum* isolated from spring wheat and weeds have the potential to produce enniatins and moniliformin in spring wheat. Each type of weed can host different *Fusarium* species and strains that produce completely different mycotoxins. Therefore, the distribution of mycotoxins in spring wheat grain may depend more on the *Fusarium* species or strains that infect the weeds than on the pathogen’s host plant species. The predominance of specific mycotoxins in cereals depends on the year’s weather conditions and the diversity of *Fusarium* species present in the field.

## 1. Introduction

*Fusarium* spp. are considered to be among the pathogens that pose the greatest risk to crops and other plants due to the high economic losses incurred in terms of yield reductions and the risk of mycotoxin production [1]. One of the characteristics of the *Fusarium* species’ Fusarium head blight (FHB) is represented by their ability to biosynthesize various mycotoxins [2,3,4]. The primary sources of FHB infection may include crop residues left in a field from the previous season [5,6]. In recent years, weeds have also been perceived as reservoirs of fungal spores and sources of disease infection, as they emerge together with a crop during the growing season and many of them are resistant to herbicides [7]. Weeds can grow over several field seasons and provide a habitat for the overwintering and survival of *Fusarium* species [8,9]. *Fusarium* fungi produce many types of mycotoxins, the distribution of which also varies [10]. *Fusarium graminearum* is considered to be the most important causal agent of FHB in wheat in many regions, and it is locally dominant and can occur and develop in some hosts [11,12,13,14].

*Fusarium avenaceum* is widespread worldwide, well-adapted to cold regions such as Northern Europe and Canada, and is the main causal agent of FHB [15,16,17]. *F. avenaceum* has also been reported as a common saprophyte and pathogen in warmer regions, but the emergence of competing species may limit the distribution of this species and its related mycotoxins [15].

The current discourse revolves around the emergence of *Fusarium* mycotoxins, which can now be detected at considerable levels in crops worldwide due to alterations in climate and fungal biota. The main members of this group are enniatins (ENN), beauvericin (BEA), and moniliformin (MON) [17].

Enniatins have been reported in Europe as contaminants of wheat, rye, oats, barley, and sorghum [17,18,19], with *F. avenaceum* as the predominant species in the cereal grains where enniatins are detected [20]. *F. avenaceum* is a prevalent plant pathogen and soil saprophyte with a wide host range [21].

As of now, there are no regulatory thresholds in place for MON. The European Food Safety Authority (EFSA) has indicated that additional studies on its toxicity are necessary and has recommended the gathering of further data on the prevalence of MON [22]. The detection of MON contamination in cereals from both Scandinavian and Southern European countries indicates that it can be synthesized by various *Fusarium* species under diverse climatic conditions [19,23,24,25].

Regrettably, our understanding of the bioavailability and production of numerous lesser-known mycotoxins, such as the extensive enniatin family, remains limited. In this study, we present occurrence data for mycotoxin production under field conditions in spring wheat that was spray-inoculated with *F. graminearum* and *F. avenaceum* strains from weeds.

## 2. Results

In this study, specific mycotoxins were detected in between 0% and 100% of spring wheat samples. In some cases, the grains were contaminated with more than one mycotoxin. The predominant mycotoxins detected from the samples field-inoculated with the *F. avenaceum* strains were enniatin B (ENN B) (100%), enniatin B1 (ENN B1) (100%), moniliformin (MON) (100%), enniatin A (ENN A) (98%), and enniatin A1 (ENN A1) (67%) (Table 1). The predominant mycotoxins detected in the samples field-inoculated with the *F. graminearum* strains were deoxynivalenol (DON) (100%), 3-acetyl-deoxynivalenol (3-ADON) (100%), 15-acetyl-deoxynivalenol (15-ADON) (84%), and nivalenol (NIV) (96%). The concentration range of these mycotoxins is presented in Table 1. We note that the highest recorded concentrations of DON exceeded 84,000 µg kg^−1^, which was 67 times higher than the regulatory limit [26].

Of the 24 strains tested for mycotoxin production, 12 were *F. avenaceum* strains and 12 were *F. graminearum* strains. In spring wheat inoculated with *F. graminearum*, DON was produced by all strains at high concentrations (31,849 µg kg^−1^, on average). In wheat inoculated with *F. avenaceum* strains, DON was detected at low levels (89 µg kg^−1^, on average). All 12 strains of *F. graminearum* also produced 3-ADON (334 µg kg^−1^, on average) and NIV (up to 39 µg kg^−1^). Furthermore, 15-ADON was produced by 10 of the 12 *F. graminearum* strains (average of 1192 µg kg^−1^ among the positive samples) and zearalenone (ZEA) was produced by 9 of the 12 *F. graminearum* strains (up to 28 µg kg^−1^). ENN B was detected in 2 of the 12 wheat samples inoculated with *F. graminearum* (up to 39 µg kg^−1^), and ENN B1 was found in 4 of the 12 samples inoculated with *F. graminearum* (up to 44 µg kg^−1^).

All 12 strains of *F. avenaceum* produced ENN A, ENN B, ENN B1, and MON, with average concentrations among the positive samples of 23, 585, 299, and 470 µg kg^−1^, respectively. Eight of the twelve *F. avenaceum* strains produced ENN A1 (up to 17 µg kg^−1^). Traces of the HT-2 toxin were also detected in 6 of the 12 samples inoculated with *F. avenaceum* (up to 23 µg kg^−1^) and in 4 of the 12 samples inoculated with *F. graminearum* (up to 20 µg kg^−1^).

**Figure 1:**
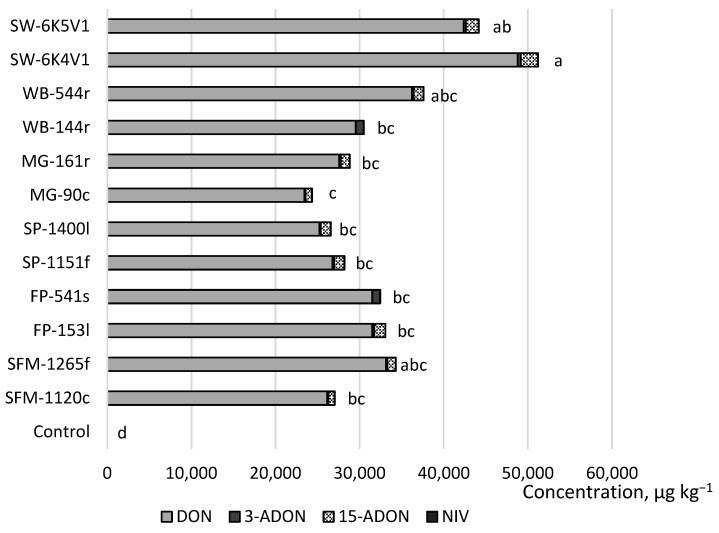
The sum of trichothecene B production potential and concentrations in spring wheat spray-inoculated with *F. graminearum* in comparison with a water control sample at field conditions. SW, spring wheat; WB, wild buckwheat; MG, meadow grass; SP, shepherd’s purse; FP, field pansy; SFM, scentless false mayweed. (Letters a–d show statistically significant differences between variables, *p* < 0.05).

Regarding the capacity for mycotoxin synthesis, all strains of *F. graminearum* that were obtained from weeds and spring wheat were determined to have the potential to produce trichothecenes, including DON, 3-ADON, 15-ADON, and NIV in spring wheat. Figure 1 shows the sum of the trichothecene production potential and concentrations in comparison to that of the water control sample. Statistically significant amounts of trichothecenes were produced by all *F. graminearum* strains isolated from weeds compared to the control. Spring wheat (SW-6K4V1) strains produced the highest combined concentrations of DON, 3-ADON, 15-ADON, and NIV. Among all strains, only one of the wild buckwheat (WB-144r) and one of the field pansy (FP-541s) samples did not produce 15-ADON in spring wheat. The spring wheat strain SW-6K4V1 produced the highest concentration of 15-ADON. The wild buckwheat strain WB-144r produced the highest concentration of 3-ADON. The most prominent producers of NIV were the strains isolated from spring wheat. All strains produced DON at particularly high concentrations. The spring wheat strain SW-6K4V1 produced the highest concentration of DON. We did not find statistical differences between the strains from the same host plants.

**Figure 2:**
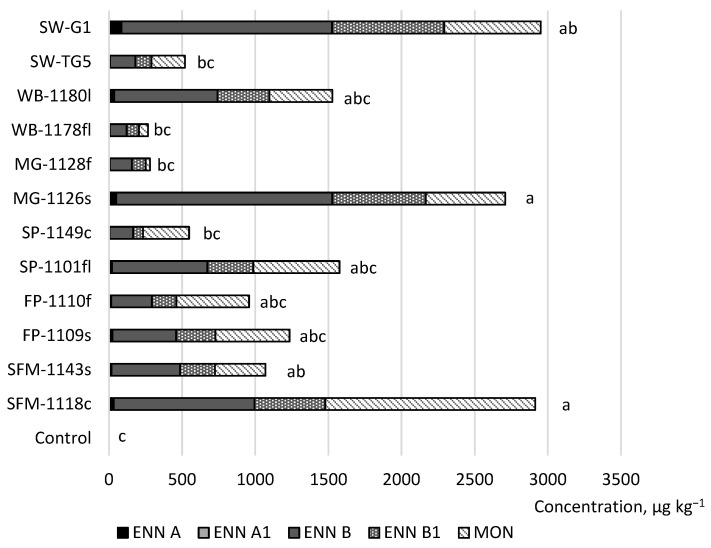
The sum of the mycotoxin production potential and concentrations observed at field conditions in spring wheat spray-inoculated with *F. avenaceum* in comparison with the water control sample. SW, spring wheat; WB, wild buckwheat; MG, meadow grass; SP, shepherd’s purse; FP, field pansy; SFM, scentless false mayweed. (Letters a–c show statistically significant differences between variables, *p* < 0.05).

The investigation discovered that all the strains of *F. avenaceum* that were isolated from weeds and spring wheat had the potential to generate enniatins and moniliformin in spring wheat, with the potential for mycotoxin synthesis varying by strain. Figure 2 shows the production potential for the sum of the ENNs and MON found and the observed concentrations in comparison with the control sample. Compared to the control, statistically significant amounts of ENNs and MON were produced by the *F. avenaceum* strains isolated from weeds (two strains) and spring wheat (one strain). One spring wheat strain (SW-G1) produced the highest concentration of the sum of ENN A, ENN A1, ENN B, ENN B1, and MON.

All the strains isolated from *F. avenaceum* produced ENN A. The spring wheat strain SW-G1 produced the highest concentration of ENN A. The shepherd’s purse strain SP-1149s produced the lowest concentration of this mycotoxin group.

One strain of shepherd’s purse (SP-1149s), one of meadow grass (MG-1128f)*,* one of wild buckwheat (WB-1178fl), and one of spring wheat (SW-TG5) did not produce ENN A1 in spring wheat. The spring wheat strain SW-G1 produced the highest concentration of ENN A. Although the ENN A concentrations were very low, they were detected in many samples.

In our study, all the *F. avenaceum* strains also produced ENN B and ENN B1. The meadow grass strain MG-1126s produced the highest concentration of ENN B. The wild buckwheat strain WB-1178fl produced the lowest concentration of this mycotoxin group. The higher ENN B producers were the strains isolated from meadow grass (MG-1126s) and spring wheat (SW-G1).

Remarkably, the meadow grass strains showed statistical differences between strains from the same host plants. We did not find statistical differences between the other strains from the same host plants.

Among the samples inoculated with the *F. avenaceum* strains in the field, enniatin B was the toxin found in the highest quantity, while ENN B1, ENN A, and ENN A1 followed in descending order. The samples inoculated with the *F. avenaceum* strain isolated from scentless false mayweed (SFM-1118c) exhibited greater concentrations of MON compared to the control.

In this study, we found that ENN A and ENN B1 co-occurred with DON in the wheat samples field-inoculated with the *F. avenaceum* strains.

Zearalenone (ZEA), neosolaniol (NEO), T-2, and HT-2 were not detected at all or were present at trace concentrations, mostly below the limit of detection (<LOD), in all tested samples.

The concentrations of the different mycotoxins were evaluated for their correlation using Pearson correlation tests. The correlation between moniliformin and the enniatins was examined, resulting in Pearson’s correlation coefficients of 0.98 and 0.63 for the associations between ENN BENN B1 and MON, respectively. The correlation coefficient 0.65 was calculated for the correlation between MON and ENN B1. The correlation between deoxynivalenol, the enniatins, and moniliform was also investigated. The correlation coefficients −0.38, −0.49, and −0.50 were calculated for the correlations between DON and MON, ENN B, and ENN B1, respectively. The correlation coefficient −0.50 was calculated for the correlation between DON and the sum of the enniatins (A, A1, B, and B1).

## 3. Discussion

This study compared the mycotoxin contamination potential among various *Fusarium* species obtained from different host plant groups, including weeds and *Triticum*. Previous data have indicated that *Fusarium* isolates from various host plants can produce FHB disease with differing severity [27,28]. Weeds often become a source of pathogens when there are no significant host plants nearby [29]. It is known that *F. graminearum* strains from alternative host plants are potential producers of trichothecenes [4]. In previous research by Krnjaja et al. [30], a quantitative analysis indicated a significant potential for producing DON among the identified strains isolated from *Triticum* at amounts that exceeded 20,000 µg kg^−1^. In our study, various *F. graminearum* strains isolated from weeds and *Triticum* also were characterized as having high toxigenic potential towards the production of trichothecenes. Various *F. graminearum* strains isolated from weeds produced DON at average levels of more than 29,000 µg kg^−1^. *F. graminearum* strains isolated from spring wheat produced DON with an average concentration of more than 45,000 µg kg^−1^. Stanković et al. [31] found significant levels of DON in *F. graminearum* strains isolated from wheat grains collected from different regions in Serbia. Their study showed that the high levels of DON, ranging from 160 to 45,260 µg kg^−1^, varied depending on the region. Obradović et al. [32] reported wheat samples with high DON levels ranging from 23,800 to 88,700 µg kg^−1^. According to Gerling et al. [33], the highest levels of DON and ZEN mycotoxins were found in the sampling sites closest to the *Fusarium* spp.-infested grass strips. Other studies have shown that DON and NIV can be detected at relatively low frequencies and concentrations in weed samples [34,35]. However, in the study by Dong et al. [34], DON and NIV were detected in >50% of the samples, and the concentrations were 1468 and 303 µg kg^−1^, respectively. NIV was also detected in 96% of the samples in our study, but the maximum concentration was 39 µg kg^−1^. In addition, in our study, *F. graminearum* strains isolated from weeds produced significant amounts of 15-ADON, with an average concentration of more than 800 µg kg^−1^. In contrast, strains isolated from spring wheat produced double the amount of 15-ADON, with an average concentration of more than 1700 µg kg^−1^. *F. graminearum* is considered to be the most important causative agent of FHB and the most aggressive producer of DON. Under favorable conditions, it can spread up to 33 m into a wheat field from a source of infection, such as wild grasses or weeds [33].

In the few field-inoculated grain samples with *F. graminearum* strains, ENN B and ENN B1 were detected but not quantified. Moniliformin was not detected in the grain samples field-inoculated with the *F. graminearum* strains. Jestoi et al. [36] detected MON only in a sample that was contaminated with *F. avenaceum*.

In our study, in contrast to *F. graminearum*, the *F. avenaceum* strains isolated from weeds and spring wheat produced higher levels of ENNs and MON. Only low levels of DON were detected in the grain samples field-inoculated with *F. avenaceum* strains, pointing to the significance of wheat infestation with DON producers from the environment. This was also confirmed by the traces of mycotoxins detected in the water control samples.

Bertuzzi et al. [37] found that the level of MON exceeded that of DON in their study. The co-occurrence of these toxins was observed in 43.7% of the samples, but no significant correlation was established. This led to the assumption that the toxins were produced by different species of *Fusarium*. In our study, the correlation between the concentrations of DON and MON was also insignificant. These findings corroborated the results of Beccari et al. [38], who investigated the various Fusarium species present in Italian durum wheat and assessed their potential to produce mycotoxins in vitro. Specifically, *F. avenaceum* strains were found to produce high levels of MON, while *F. graminearum* strains were predominantly associated with DON production. In a study from France, ENN B was the most frequent (68%) of the total enniatin content, followed by ENN B1 (22%), ENN A1 (7%), and ENN A (3%) [39]. In good agreement with our findings, *F. avenaceum* was the most prolific producer of ENNs, including ENN B (62% of the total enniatin content), followed by ENN B1 (36%), ENN A (2%), and ENN A1 (0%).

Thus, weed control is crucial for disease prevention in crops, especially pertaining to fungal invasion. Weeds can serve as a source of fungal inoculum and compete with crops for water and nutrients, thereby weakening them and contributing to contamination [40]. Grasses should also be considered as a source of *Fusarium* infection, especially after rainfall during flowering or when a field is irrigated [33]. Consequently, weed control measures should be consistently implemented.

## 4. Conclusions

This study has revealed that different species and strains of *Fusarium* can occur in the same weed or other host plants, producing completely different mycotoxins. Therefore, the distribution of mycotoxins in spring wheat grain may depend more on the *Fusarium* species and strains that infect weeds than on the pathogen’s host plant species.

Some *Fusarium avenaceum* strains from weeds may have more potential for MON production than those from spring wheat.

The specific mycotoxin types dominating in cereals depend on the year’s climatic conditions and the diversity of *Fusarium* species present in the field environment. If the climatic conditions are favorable for *F. avenaceum*, more enniatins and moniliformin are likely to be produced. If the climatic conditions are more favorable for *F. graminearum*, then more trichothecenes B will be produced.

In integrated pathogen control, weed control should be a key focus, as weeds may not show disease symptoms but may be a potential source of *Fusarium* infections in spring wheat. This leads to reductions in grain yield and grain quality and a high risk of mycotoxins in the grain. In the future, attention should be paid not only to the regulated mycotoxins but also to newly emerging modified mycotoxins, the occurrence of which may be influenced by the presence of weeds in crops.

## 5. Materials and Methods

### 5.1. Sample Collection

From 2015 to 2016, asymptomatic weeds were collected in fields situated in Central Lithuania (55°23′50″ N, 23°51′40″ E) and *F. graminearum* and *F. avenaceum* strains were isolated [41]. An inoculation procedure was conducted on heads of the spring wheat cultivar ‘KWS Chamsin’ in 2019 at the Lithuanian Research Centre for Agriculture and Forestry in experimental fields. During mid-flowering, the main *Fusarium* pathogens responsible for Fusarium head blight (FHB), including *F. avenaceum* and *F. graminearum*, were isolated from the internal tissues of asymptomatic weeds and were used to inoculate the ears of spring wheat [28].

A total of 12 *F. graminearum* and 12 *F. avenaceum* strains were isolated, comprising 10 *F. graminearum* and 10 *F. avenaceum* strains from asymptomatic weeds (*Capsella bursa-pastoris*, *Fallopia convolvulus*, *Poa annua*, *Tripleurospermum inodorum*, and *Viola arvensis*), 2 *F. graminearum* from the primary host plant spring wheat (*Triticum aestivum*), and 2 *F. avenaceum* from the primary host plant spring wheat (*Triticum aestivum*). Under field conditions, these strains were evaluated for their capacity to produce mycotoxins in grains of spring wheat. A total of 25 treatments were tested in four replicates. The control samples were inoculated with sterile distilled water. The study scheme is presented in Table 2. The wheat head inoculation procedure was carried out following the methodology described in prior research studies [28,41].

### 5.2. Sample Preparation for Mycotoxin Analyses

We used 50 mL polypropylene (PP) tubes to extract the ground samples, which weighed 2.50 ± 0.01 g, with a mixture of deionized water (10 mL), acetonitrile (10 mL), and formic acid (20 µL) on a mechanical shaker for 10 min. Following the addition of the QuEChERS salt mixture, the samples were shaken for 10 min on a mechanical shaker, then centrifuged for 10 min at 4000 rpm at room temperature. The supernatants were transferred to 15 mL PP tubes, which were then placed in an ultra-low temperature freezer for 15 min at −80 °C. After removal from the freezer, the tubes were immediately centrifuged again at 4000 rpm for 10 min at a temperature of 10 °C.

We used 15 mL PP tubes to transfer 3 mL portions of the extracts, which were then evaporated to dryness under a slow nitrogen stream at 50 °C. After evaporation, 100 μL of a 0.1% solution of formic acid in acetonitrile: water (1:1) was added to the samples, which were shaken on a Vortex mixer. Subsequently, 250 µL of a 0.1% aqueous formic acid solution was added. Then, 0.22 µm polyvinylidene difluoride membrane filters (PVDF) were used to filter the extracts and then they were centrifuged for 10 min at room temperature at 3000 rpm. Matrix-match calibration was used for quantification. A blank sample extract was used as the matrix component. For samples with mycotoxin concentrations exceeding the highest calibration level, the sample preparations were repeated, but instead of evaporation, the extracts were diluted and quantified using external solution calibration.

In the case of MON, after sample freezing and centrifugation, a 1 mL aliquot was taken and evaporated. The residue that was dried was then dissolved again in 100 μL of acetonitrile.

### 5.3. Method of Analysis

An HPLC analysis was conducted using an UltiMate 3000 instrument (Thermo Fisher Scientific, Waltham, MA, USA) equipped with a Thermo Scientific TSQ Quantiva MS/MS detector (Thermo Fisher Scientific, Waltham, MA, USA). Separation was carried out on a Phenomenex Luna C18 reversed-phase analytical column (150 × 2.0 mm, 3 µm) for the toxins DON, 3-ADON, 15-ADON, NIV, NEO, T-2, HT-2, ZEA, ENN A, ENN A1, ENN B, and ENN B1, and a Phenomenex Luna HILIC analytical column (100 × 3.0 mm, 3 µm) was used for MON. The autosampler was set at 4 °C and the column temperature was set at 40 °C. The sample injection volume was 25 µL for DON, 3-ADON, 15-ADON, NIV, NEO, T-2, HT-2, ZEA, ENN A, ENN A1, ENN B, and ENN B1, and 5 µL for MON. Ion monitoring was conducted in both positive and negative ion modes using selected reaction monitoring (SRM) (Table 3). The instrument settings included a spray voltage of 3.5 kV (positive ion mode) and 2.5 kV (negative ion mode), a vaporizer temperature of 350 °C, an ion transfer temperature of 300 °C, sheath gas at 55 arbitrary units (arb), auxiliary gas at 25 arb, and sweep gas at 5 arb. Data processing was performed with TraceFinder and Xcalibur™ software (Thermo Fisher Scientific, Waltham, MA, USA) [42]. The composition of Phase A was 0.1% formic acid and 0.5 mM ammonium acetate in water, while Phase B was composed of 0.1% formic acid and 0.5 mM ammonium acetate in acetonitrile.

### 5.4. Method Validation

To evaluate the linearity, the standard mycotoxin mixtures were spiked into blanks to create five-point calibration curves. The least-squares regression method was used to calculate the slope and determination coefficients (R2) of the calibration curves, which were considered a good fit if they were equal to or greater than 0.99. For quality control purposes, the blank samples were spiked with mycotoxin standards at the following concentration levels: 10, 50, and 100 µg kg^−1^ for DON, 3-ADON, 15-ADON, NIV, NEO, T-2, HT-2, ZEA, and the enniatins (A, A1, B, and B1) and 100 and 800 µg kg^−1^ for MON. Standard deviations (Sn) were obtained from 6 replicates of the spiked samples at the lowest (10 µg/kg) validated levels for each compound. Limit of detection (LOD) and limit of quantification (LOQ) levels were obtained during the validation procedure, and the following formulas were used for the calculations: LOD = 3.3 · Sn (6), LOQ = 10 · Sn (6) [43].

Table 3 displays the results of the method validation, which included the analysis of five replicates at each of the three spiking levels to validate the precision and accuracy of the method.

### 5.5. Meteorological Conditions

In Lithuania, the prevailing meteorological conditions are conducive to the growth of Fusarium fungi, which cause plant diseases and produce mycotoxins, leading to significant damage. On 17 June 2019, spring wheat heads were inoculated with *F. graminearum* and *F. avenaceum* isolated from weeds. Meteorological data were taken from the central Lithuania meteorological station (55°23′49″ N, 23°51′55″ E, Kedainiai district). The year 2019 had high humidity and warm temperatures during the wheat flowering and seed filling stages, which led to an elevated risk of Fusarium head blight (FHB) and subsequent mycotoxin production in harvested grains. More detailed information on meteorological conditions is available in the study by Matelionienė et al. [28].

### 5.6. Statistical Analysis

Statistical analysis was performed using SAS Enterprise Guide 7.1 to ensure data reliability. The data scatter and differences between data averages were evaluated using a one-way analysis of variance (ANOVA) package, and significant differences between the two samples were determined using Duncan’s criterion. The significance level was set at *p* < 0.05.

## Figures and Tables

**Table 1:** Occurrence of mycotoxins in field-inoculated spring wheat grain samples according to *Fusarium* species.

Mycotoxin	*F. avenaceum*				*F. graminearum*			
	Positive (%)	Minimum (µg kg^−1^)	Maximum (µg kg^−1^)	Average (µg kg^−1^)	Positive (%)	Minimum (µg kg^−1^)	Maximum (µg kg^−1^)	Average (µg kg^−1^)
DON *	98	<5.4	636	89	100	5120	84,319	31,849
NIV *	0	<11.8	<11.8	<11.8	96	<11.8	39	14
3-ADON *	0	<11	<11	<11	100	27	1245	334
15-ADON *	0	<42	<42	<42	84	<42	3915	994
ZEA *	0	<10.3	<10.3	<10.3	52	<10.3	28	4
NEO *	0	<4.6	<4.6	<4.6	0	<4.6	<4.6	<4.6
ENN A *	98	<5.7	170	23	2	<5.7	1	0
ENN A1 *	67	<5.3	17	2	0	<5.3	<5.3	<5.3
ENN B *	100	1	2749	585	6	<10.7	39	1
ENN B1 *	100	18	1541	299	10	<9.3	44	4
T-2 *	2	<6.1	2	0	4	<6.1	2	0
HT-2 *	17	<7.4	23	1	12	<7.4	20	1
MON *	100	5	4653	470	0	<2	<2	<2

* DON, deoxynivalenol; NIV, nivalenol; 3-ADON, 3-acetyl-deoxynivalenol; 15-ADON, 15-acetyl-deoxynivalenol; ZEA, zearalenone; NEO, neosolaniol; ENN A, enniatin A; ENN A1, enniatin A1; ENN B, enniatin B; ENN B1, enniatin B1; T-2, T-2 toxin; HT-2, HT-2 toxin; MON, moniliformin.

**Table 2:** The study’s design.

Treatment No.	Host Plant	*F. avenaceum* Strain Code	*F. graminearum* Strain Code
1	Spring wheat (*Triticum aestivum*)	SW-G1	SW-6K5V1
2		SW-TG5	SW-6K4V1
3	Wild buckwheat (*Fallopia convolvulus* (L.) Löve)	WB-1180l	WB-544r
4		WB-1178fl	WB-144r
5	Meadow grass (*Poa annua* L.)	MG-1128f	MG-161r
6		MG-1126s	MG-90c
7	Shepherd’s purse (*Capsella bursa-pastoris* (L.) Medik.)	SP-1149c	SP-1400l
8		SP-1101fl	SP-1151f
9	Field pansy (*Viola arvensis* Murray)	FP-1110f	FP-541s
10		FP-1109s	FP-153l
11	Scentless false mayweed *(Tripleurospermum inodorum* (L.) Sch.)	SFM-1143s	SFM-1265f
12		SFM-1118c	SFM-1120c
13	Control	Sterile distilled water	

SW, spring wheat; WB, wild buckwheat; MG, meadow grass; SP, shepherd’s purse; FP, field pansy; SFM, scentless false mayweed; c, f, fl, l, r, and s, isolates obtained from crowns (c), fruits (f), flowers (fl), leaves (l), roots (r), and stems (s).

**Table 3:** Parameters for the validation of the chromatography method.

Validation Parameters												
Mycotoxin	Retention Time (min)	Polarity	LOD * (µg kg^−1^)	LOQ * (µg kg^−1^)	Linear Range (µg kg^−1^)	R^2^ *	Accuracy (Deviation from the Theoretical Value (%))			Precision (RSD * (%))		
							Level of Spiked Samples (µg kg^−1^)					
							10	50	100	10	50	100
NIV	3.4	Positive	3.9	11.8	10–250	0.9992	3	−5	2	11	6	2
DON	5.9	Positive	1.8	5.4	10–500	0.9993	−2	−6	−6	6	3	4
NEO	7.9	Positive	1.5	4.6	10–100	0.9994	10	−1	−4	4	3	3
15-ADON	8.4	Positive	14	42	10–500	0.9988	x	4	2	x	8	2
3-ADON	8.6	Positive	3.6	11	10–500	0.9991	−24	−11	−9	14	14	11
HT-2	11.2	Positive	2.4	7.4	10–500	0.9998	8	−5	8	7	2	2
T-2	11.9	Positive	2.0	6.1	10–100	0.9989	−14	−6	−7	7	5	4
ZEA	12.5	Negative	3.4	10.3	10–500	0.9992	19	−4	1	9	7	6
ENN B	14.1	Positive	3.5	10.7	10–100	0.9972	−29	0	−7	14	4	4
ENN B1	14.1	Positive	3.0	9.3	10–500	0.9998	−15	4	−7	11	7	5
ENN A	14.3	Positive	1.9	5.7	10–500	0.9998	−15	1	−11	7	3	5
ENN A1	14.4	Positive	1.7	5.3	10–500	0.9998	−5	−1	−14	6	7	8
							Level of spiked samples (µg kg^−1^)					
								100	800		100	800
MON	11	Negative	0.6	2	50–1000	0.9974		7	4		23	9

* LOD, limit of detection; LOQ, limit of quantification; R^2^, coefficient of determination; RSD, relative standard deviation.

## Data Availability

The data presented in this study are available in this article.
